# Mild Attenuation of the Pulmonary Inflammatory Response in a Mouse Model of Hereditary Hemochromatosis Type 4

**DOI:** 10.3389/fphys.2020.589351

**Published:** 2021-01-13

**Authors:** Oriana Marques, Joana Neves, Natalie K. Horvat, Sandro Altamura, Martina U. Muckenthaler

**Affiliations:** ^1^Department of Pediatric Oncology, Hematology, Immunology and Pulmonology, University Hospital Heidelberg, Heidelberg, Germany; ^2^Molecular Medicine Partnership Unit, University of Heidelberg, Heidelberg, Germany; ^3^Translational Lung Research Center Heidelberg (TLRC), German Center for Lung Research (DZL), University of Heidelberg, Heidelberg, Germany; ^4^European Molecular Biology Laboratory, Heidelberg, Germany

**Keywords:** lung, inflammation, iron, alveolar macrophages, neutrophils, ferroportin

## Abstract

The respiratory tract is constantly exposed to pathogens that require iron for proliferation and virulence. Pulmonary iron levels are increased in several lung diseases and associated with increased susceptibility to infections. However, regulation of lung iron homeostasis and its cross talk to pulmonary immune responses are largely unexplored. Here we investigated how increased lung iron levels affect the early pulmonary inflammatory response. We induced acute local pulmonary inflammation via aerosolized LPS in a mouse model of hereditary hemochromatosis type 4 (*Slc40a1*^C326S/C326S^), which is hallmarked by systemic and pulmonary iron accumulation, specifically in alveolar macrophages. We show that *Slc40a1*^C326S/C326S^ mice display a mild attenuation in the LPS-induced pulmonary inflammatory response, with a reduced upregulation of some pro-inflammatory cytokines and chemokines. Despite mildly reduced cytokine levels, there is no short-term impairment in the recruitment of neutrophils into the bronchoalveolar space. These data suggest that increased pulmonary iron levels do not strongly alter the acute inflammatory response of the lung.

## Introduction

Iron is essential for the proliferation and virulence of most pathogens. To reduce iron availability for extracellular pathogens in the plasma, the host sequesters iron intracellularly in reticuloendothelial macrophages (Ganz and Nemeth, 2009). Two different mechanisms contribute to inflammation-associated reduction of plasma iron levels – a process termed hypoferremia. On the one hand, inflammatory signals (e.g., Interleukin [IL] 6) stimulate the expression of the hepatic hormone hepcidin, which binds to the cellular iron exporter ferroportin, causing its proteolytic degradation (Nemeth et al., 2004a,Nemeth et al., 2004b). Since ferroportin is predominantly expressed in cells that handle major iron flows, such as macrophages and duodenal enterocytes, hepcidin binding causes a decrease in iron release from these iron-exporting cell types and hypoferremia develops. On the other hand, ferroportin expression in macrophages is also downregulated at the transcriptional level via TLR4- and TLR2/TLR6- signaling pathways (Liu et al., 2005; Peyssonnaux et al., 2006; Guida et al., 2015). The resulting decrease in iron export reduces plasma iron levels independently of hepcidin (Guida et al., 2015). These responses reveal an active and important cross-talk between the control of systemic iron metabolism and immune responses and demonstrate how iron homeostasis is altered by inflammation. However, the impact of altered iron homeostasis on the immune response is largely unexplored.

The aim of this study was to investigate whether increased systemic and lung iron levels affect the early pulmonary inflammatory response. The lung is constantly exposed to inhaled pathogens. Therefore iron levels must be tightly controlled to limit availability to these invading microorganisms. Interestingly, increased lung iron levels have been reported in several lung diseases and are associated with increased susceptibility to pulmonary infections (Reid et al., 2007; Ali et al., 2017). As infectious complications are often the primary cause or opportunistic side-effects in respiratory syndromes it is of clinical relevance to understand the role of the host iron status (Zhang et al., 2019).

As a mouse model for iron overload we analyzed the *Slc40a1*^C326S/C326S^ mouse, a disease model for hereditary hemochromatosis type 4, in which a point mutation in the murine ferroportin protein confers resistance to hepcidin binding (Altamura et al., 2014). This contrasts the situation in iron-loaded wild-type (WT) mice, where increased hepcidin levels attenuate iron fluxes by triggering hepcidin-mediated ferroportin internalization and degradation (Nemeth et al., 2004b). As a consequence of the C326S mutation, ferroportin is stabilized at the cell membrane and there is unregulated iron export from enterocytes and macrophages to the bloodstream. As a result plasma iron levels increase and iron accumulates in several organs, such as the liver, heart, and pancreas, while iron is depleted in enterocytes, as well as splenic and liver macrophages (Altamura et al., 2014). We have previously reported that iron also accumulates in the lung of *Slc40a1*^C326S/C326S^ mice, particularly in alveolar macrophages, which play a key role in the pulmonary inflammatory response against inhaled pathogens (Neves et al., 2017).

In this study, we induced acute inflammation in the lung of *Slc40a1*^C326S/C326S^ mice by inhalation of LPS, as a model of gram-negative bacterial infection. This approach allowed us to exclude confounding factors present in multifactorial diseases and therefore to directly address the role of iron in pulmonary inflammation. Our results show that increased systemic and/or lung iron levels cause a mild attenuation in the pulmonary acute inflammatory response, without significantly affecting the LPS-induced recruitment of neutrophils to the alveolar space.

## Materials and Methods

### Mice

14 week-old female *Slc40a1*^C326S/C326S^ mice were maintained on a pure C57BL/6N genetic background (>99.9% congenic). To avoid variability of iron-related parameters during LPS treatment analyses were restricted to female mice. Of note, iron-related parameters (e.g., hepcidin levels) can vary between sexes, although differential iron accumulation in the lung was not reported (Latour et al., 2014). As controls, age- and gender-matched WT C57BL/6N mice born and maintained in the same breeding facility were analyzed. Mice were housed in the Heidelberg University animal facility under constant light-dark cycle and maintained on a standard mouse diet (LASQCdiet Rod18-A – LASvendi) containing 200 ppm iron with *ad libitum* access to food and water. All mouse breeding and animal experiments were approved by the Regierungspräsidium Karlsruhe (projects G-39/16 and G-41/16, respectively).

### LPS Nebulization

*E. coli* lipopolysaccharides (LPS, serotype O111:B4; Sigma) was delivered to the lung using a nebulizer connected to a chamber (volume of 1L) – this method was adapted from Roos et al. (2014). Dissolved LPS (400 μg/mL) was aerosolized for 2 min, with a flow of 250 μL/min. Mice were exposed to nebulized LPS in total for 5 min (2 min of continuous flow plus 3 min after the nebulizer was turned off). Four hours after LPS treatment, mice were anesthetized via intraperitoneal injection of a combination of ketamine and xylazine (120 and 16 mg/kg, respectively) and sacrificed by exsanguination. Before harvesting, the lung was perfused via the heart with 10 mL of Phosphate Buffered Saline (PBS).

### Bronchoalveolar Lavage (BAL)

A median sternotomy was performed, the trachea cannulated and the left mainstem bronchus ligated, and the right lung was lavaged with PBS. The cells were collected by centrifugation and cytospined for staining with May-Grünwald-Giemsa (Merck) or Perls’ Prussian Blue (Neves et al., 2017). Alternatively, the cells were analyzed by flow cytometry and total numbers were determined by counting 0.4% Trypan Blue (Sigma)-stained cells in a hemocytometer.

### Hematoxylin and Eosin Stain

Paraffin lung sections (2 μm) were rehydrated and stained for 6 min with Mayer’s Hematoxylin (Sigma Aldrich). After washing in water and rinsing in HCl/EtOH, sections were stained for 1 min with Eosin Y (Sigma Aldrich). Slides were dehydrated and mounted using Entellan (Merck).

### Flow Cytometry

Cells obtained from the BAL were stained with CD45.2, CD11c, SiglecF, and Ly6G antibodies for 30 min on ice (antibody dilutions and catalog numbers reported in Supplementary Table 1). After washing cells were analyzed by flow cytometry using the BD Accuri C6 Plus.

### RNA Extraction, Reverse Transcription, and qRT-PCR

RNA was extracted from total lung using Trizol (Life Technologies). 1 μg of total RNA was reverse transcribed in a 25 μL reaction mixture using RevertAid H Minus reverse transcriptase (Thermo Scientific) and random oligomers as primers. SYBR green qRT-PCR was performed using the StepONE Plus real-time PCR system (Applied Biosystems). mRNA expression of the gene of interest was normalized to Rpl19 and data were analyzed using the ΔΔCt method (Pfaffl, 2001). The primers used are listed in Supplementary Table 2.

### Western Blotting

Protein lysates were obtained by homogenizing snap-frozen lung tissue in radioimmunoprecipitation assay (RIPA) buffer supplemented with a protease inhibitor cocktail (cOmplete tablets EASYpack, Roche). Protein concentration was determined using the BCA assay (Thermo Scientific). 50 μg of total protein extracts were subjected to western-blot analysis with the antibodies listed in Supplementary Table 3. Western Blot images were acquired with the Vilber Lourmat Fusion-FX Chemiluminescence system. β-actin was used as loading control.

### Tissue Non-heme Iron Measurement

Lung, liver and spleen non-heme iron content were measured using the bathophenantroline method and calculated against dry weight tissue (Torrance and Bothwell, 1968).

### Statistical Analyses

Data are shown as mean ± standard error of the mean (SEM). Number of mice analyzed (n) is indicated in the graph. Statistical analyses were performed using Prism v6 (GraphPad Software). Comparisons between groups were performed using two-way ANOVA, followed by the Tukey *post-hoc* test, and *p*-values < 0.05 (^∗^), <0.01 (^∗∗^), <0.001 (^∗∗∗^), and *p* < 0.0001 (^****^) are indicated.

## Results

### Systemic and Pulmonary Iron Overload Attenuate the Expression of Some Pro-inflammatory Mediators in Response to LPS Treatment

The pulmonary inflammatory response to LPS is characterized by increased expression of multiple pro-inflammatory cytokines and chemokines, and a recruitment of immune cells, mainly neutrophils, into the bronchoalveolar space (Benesova et al., 2012). To induce an acute pulmonary inflammation, we exposed WT and *Slc40a1*^C326S/C326S^ mice, presenting increased systemic and pulmonary iron levels, namely in alveolar macrophages, ciliated airway epithelial cells, alveolar type II cells, and vascular smooth muscle cells (Altamura et al., 2014; Neves et al., 2017), to aerosolized LPS and compared their inflammatory response after 4 h. LPS instillation in WT and *Slc40a1*^C326S/C326S^ mice caused no obvious lung tissue abnormalities such as leukocyte infiltration or acute injury (Supplementary Figure 1).

**FIGURE 1:**
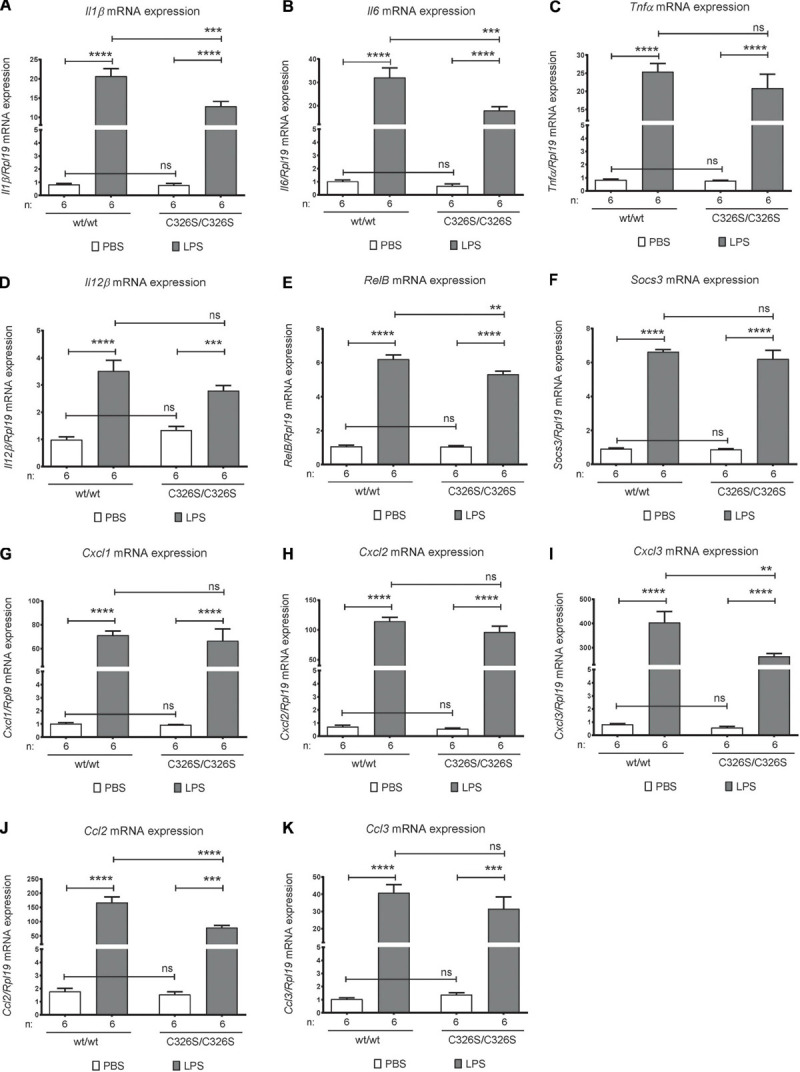
Pulmonary inflammatory response upon LPS inhalation. qRT-PCR analysis of *Il1*β **(A)**, *Il6* **(B)**, *Tnf*α **(C)**, *IL12*β **(D)**, *RelB* **(E)**, *Socs3* **(F)**, *Cxcl1* **(G)**, *Cxcl2* **(H)**, *Cxcl3* **(I)**, *Ccl2* **(J)**, and *Ccl3* **(K)** in the total lung of 14-week old female wild-type (wt/wt) and *Slc40a1*^C326S/C326S^ (C326S/C326S) mice upon PBS or LPS nebulization. Number of mice analyzed (n) is indicated in each graph. Data are reported as mean ± SEM. Two-way ANOVA: **p < 0.01; ***p < 0.001; ****p < 0.0001.

To investigate the consequences of increased iron levels on the early pulmonary inflammatory response, we analyzed the mRNA levels of inflammatory mediators in total lung. In untreated conditions there was no difference in cytokine levels in WT compared to *Slc40a1*^C326S/C326S^ mice (Figures 1A–D). Upon exposure to LPS, WT mice increase *Il1*β, *Il6*, *Tnf*α, and *Il12*β mRNA levels in the lung (Figures 1A–D). The same cytokines are also upregulated in the lung of *Slc40a1*^C326S/C326S^ mice upon LPS exposure (Figures 1A–D). However, *Il1*β and *Il6* activation in *Slc40a1*^C326S/C326S^ mice was attenuated compared to WT mice (Figures 1A,B). A similar trend was observed for *Tnf*α and *Il12*β although statistical significance was not reached.

NF-κB is a key transcription factor controlling inflammatory responses. Despite being mainly controlled by phosphorylation and nuclear translocation, one of its subunits, RELB, is also regulated at the transcriptional level upon inflammatory stimuli (Bren et al., 2001). Accordingly, we observed increased *RelB* mRNA levels in the lung of WT and *Slc40a1*^C326S/C326S^ mice upon LPS treatment (Figure 1E). Consistent with the reduced levels of some pro-inflammatory cytokines, the up-regulation of *RelB* mRNA levels was also attenuated in *Slc40a1*^C326S/C326S^ mice (Figure 1E).

SOCS3 (suppressor of cytokine signaling-3) acts as a negative feedback suppressor of cytokine signaling, in order to prevent excessive inflammation. Both WT and *Slc40a1*^C326S/C326S^ mice display equally up-regulated *Socs3* mRNA levels in the lung upon LPS exposure (Figure 1F).

Besides cytokines, chemokines (small heparin-binding proteins) also play a critical role in leukocyte recruitment during inflammation by creating a chemokine gradient toward the center of attraction (reviewed in Bhatia et al., 2012). In both WT and *Slc40a1*^C326S/C326S^ mice pulmonary mRNA levels of the chemokines associated with neutrophil recruitment, *Cxcl1* and *Cxcl2*, are markedly upregulated upon nebulization with LPS, however, no significant difference was observed between both genotypes (Figures 1G,H). On the other hand, the classical chemokine associated with monocyte recruitment, CXCL3, despite increasing in the lung after LPS nebulization in both WT and *Slc40a1*^C326S/C326S^ mice, is significantly decreased in the lungs of *Slc40a1*^C326S/C326S^ mice when compared with WT mice (Figure 1I). Consistent with the attenuated upregulation of *Cxcl3*, the mRNA levels of the monocyte migration associated chemokine *Ccl2* were also reduced in the lungs of LPS nebulized mice. No significant differences were observed for *Ccl3* between genotypes (Figures 1J,K).

### LPS-Induced Neutrophil Recruitment Into the Lungs Is Not Altered by Iron Overload in *Slc40a1*^C326S/C326S^ Mice

**FIGURE 2:**
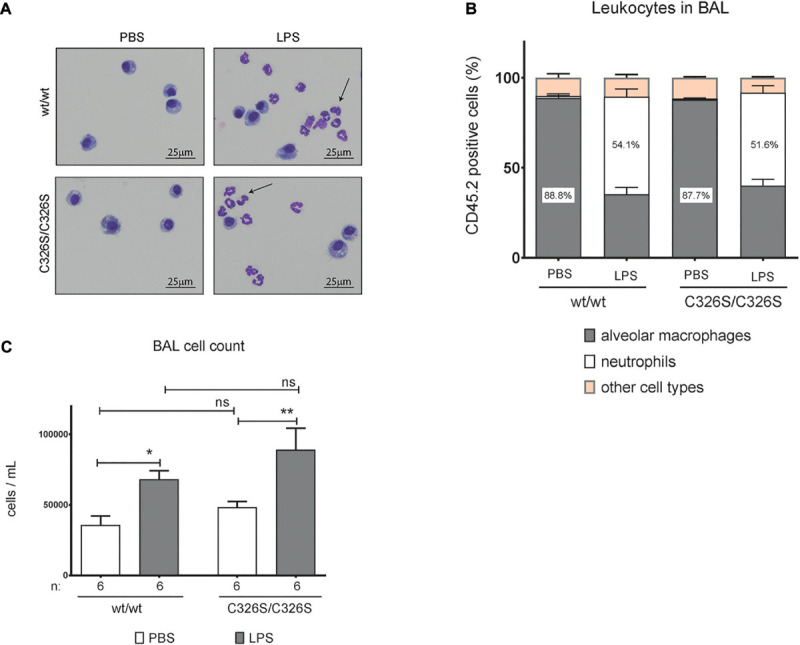
Analysis of macrophages and neutrophils isolated from the BAL of mice upon LPS treatment. **(A)** May-Grünwald-Giemsa stain of cytospin preparations of cells obtained from the BAL of 14-week old female wild-type (wt/wt) and *Slc40a1*^C326S/C326S^ (C326S/C326S) mice upon PBS or LPS nebulization, Arrows indicate neutrophils. **(B)** Flow cytometry analysis of cells obtained from the BAL of 14-week old female wild-type (wt/wt) and *Slc40a1*^C326S/C326S^ (C326S/C326S) mice upon PBS or LPS nebulization: percentage of alveolar macrophages (CD11c+ SiglecF+) and neutrophils (Ly6G+) present in the leukocyte fraction (CD45.2+). Data are reported as mean ± SEM of 6 mice per group. **(C)** Total number of cells per mL in the BAL of 14-week old female wild-type (wt/wt) and *Slc40a1*^C326S/C326S^ (C326S/C326S) mice upon PBS or LPS nebulization. Number of mice analyzed (n) is indicated in the graph. Data are reported as mean ± SEM. Two-way ANOVA: *p < 0.5; **p < 0.01.

Given the differential expression of some pro-inflammatory mediators in the lungs of LPS nebulized WT and *Slc40a1*^C326S/C326S^ mice, in particular the chemokines associated with monocyte recruitment, we questioned whether the changes are functionally relevant and cause a differential recruitment of representative cell populations to the BAL of the lung. Cells from the right lung of the mice were collected and prepared for staining with May-Grünwald-Giemsa (a commonly used technique to define and quantify blood cell populations on cytology preparations) and flow cytometry analysis. Under basal conditions, the population of cells in PBS nebulized mice is mostly comprized of alveolar macrophages, as determined by the percentage of CD45.2 positive cells simultaneously expressing CD11c and SiglecF (Figure 2B) and represented by the May-Grünwald-Giemsa stain (Figure 2A). Nebulization with LPS increases the percentage of neutrophils present in the BAL (Figure 2A) as defined by the expression of Ly6G in CD45.2 positive cells (Figure 2B). The overall percentage of alveolar macrophages and neutrophils in the BAL of *Slc40a1*^C326S/C326S^ mice, either from PBS or LPS nebulized lungs, did not significantly differ from WT mice (Two-way ANOVA adjusted *p*-value between genotypes *p* > 0.05). Furthermore, absolute cell numbers in the BAL did not differ between genotypes (Figure 2C).

### Consequence of LPS Treatment on Pulmonary Iron Homeostasis in *Slc40a1*^C326S/C326S^ Mice

**FIGURE 3:**
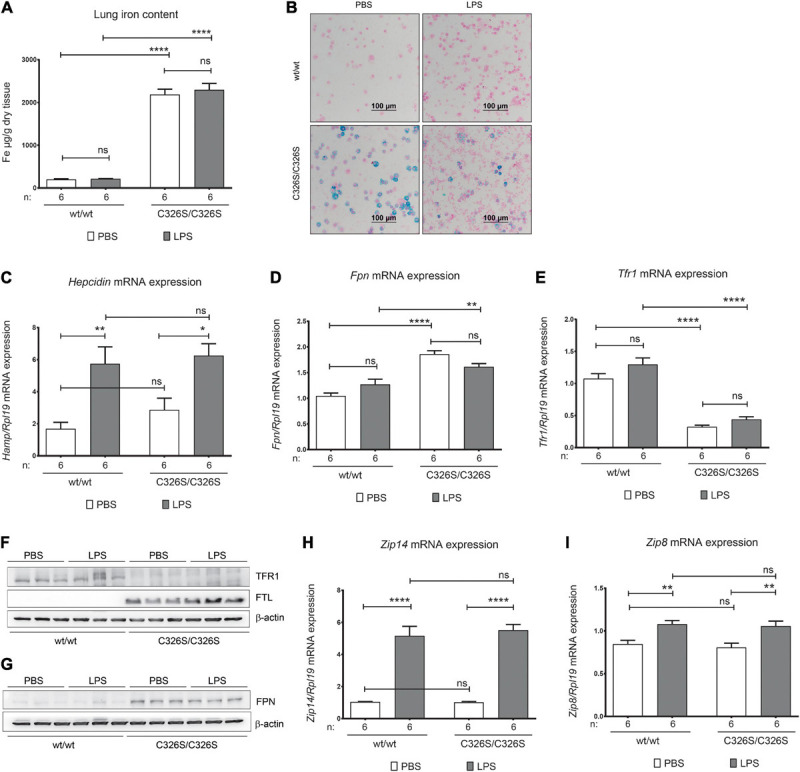
Pulmonary iron homeostasis upon LPS nebulization. **(A)** Lung non-heme iron content of 14-week old female wild-type (wt/wt) and *Slc40a1*^C326S/C326S^ (C326S/C326S) mice upon PBS or LPS nebulization. **(B)** Perl’s Prussian Blue staining of cytospin preparations of the cells isolated from the BAL fluid of 14-week old female wild-type (wt/wt) and *Slc40a1*^C326S/C326S^ (C326S/C326S) mice upon PBS or LPS nebulization. **(C–E)** qRT-PCR analysis of *hepcidin* **(C)**, *Fpn* **(D)**, and *Tfr1* **(E)** in the total lung of 14-week old female wild-type (wt/wt) and *Slc40a1*^C326S/C326S^ (C326S/C326S) mice upon PBS or LPS nebulization. **(F,G)** Western Blot analysis of TfR1, FtL and FPN protein expression in the total lung of female 14-week old wild-type (wt/wt) and *Slc40a1*^C326S/C326S^ (C326S/C326S) mice upon PBS or LPS nebulization. β-actin was used as loading control. **(H,I)** qRT-PCR analysis of *Zip14* **(G)** and *Zip8* **(H)** in the total lung of 14-week old female wild-type (wt/wt) and *Slc40a1*^C326S/C326S^ (C326S/C326S) mice upon PBS or LPS nebulization. Number of mice analyzed (n) is indicated in each graph. Data are reported as mean ± SEM. Two-way ANOVA: *p < 0.5; **p < 0.01; ****p < 0.0001.

To understand if LPS administration affects iron-related parameters in the lung of *Slc40a1*^C326S/C326S^ mice, in comparison to WT mice, we evaluated common tissue iron parameters, such as iron content as well as mRNA and protein expression of iron-related genes. 4-h following LPS administration pulmonary non-heme iron levels in WT or *Slc40a1*^C326S/C326S^ mice were not significantly altered, despite the fact that *Slc40a1*^C326S/C326S^ mice showed increased tissue iron content (Figure 3A) as previously described (Neves et al., 2017). Furthermore, no iron accumulation was observed in WT mice in response to LPS administration (Figure 3B). As previously demonstrated, the population of alveolar macrophages in *Slc40a1*^C326S/C326S^ mice displays heterogeneous iron accumulation (Figure 3B), possibly explained by differential ferroportin expression (Neves et al., 2017). LPS nebulization caused a similar increase in total lung *hepcidin* mRNA levels in WT and *Slc40a1*^C326S/C326S^ mice (Figure 3C). However, and surprisingly, no alterations in *Fpn* mRNA and protein levels were observed in both genotypes (Figures 3D,G). In addition, *Slc40a1*^C326S/C326S^ mice display decreased TfR1 mRNA and protein levels (Figures 3E,F), as well as increased FtL protein levels (Figure 3F), consistent with the iron overload phenotype and previous observations (Neves et al., 2017). This gene response is in accordance with the post-transcriptional regulation by the iron-responsive element/iron-regulatory protein (IRE/IRP) system (Muckenthaler et al., 2008). Unexpectedly, both TfR1 and FtL levels remained unaltered for both genotypes upon LPS nebulization (Figures 3E,F). Expression of the non-transferrin bound iron (NTBI) and inflammation regulated metal-ion transporters ZIP14 and ZIP8 (Galvez-Peralta et al., 2014; Muckenthaler et al., 2017) was also evaluated. Although *Zip14* and *Zip8* mRNA levels increase after LPS nebulization in the lung, no significant differences were observed between genotypes either in the steady-state or upon the inflammatory stimulus (Figures 3H,I). These findings suggest that despite increased pulmonary hepcidin levels in response to LPS inhalation iron-related parameters remain unaffected under the conditions analyzed.

## Discussion

In addition to inhaled iron-rich particles, the lung has the ability to accumulate excess iron from the blood stream (Mahowald et al., 2009; Neves et al., 2017). Pulmonary iron overload causes oxidative stress, tissue injury and ultimately impaired lung function (Neves et al., 2019). Respiratory diseases constitute a spectrum of conditions with a tremendous worldwide health burden and limited therapeutic possibilities (The Forum of International Respiratory Societies, 2017). In some acute and chronic lung diseases pulmonary iron homeostasis is altered (Ghio et al., 2003; Ghio, 2009). For example, patients with acute respiratory distress syndrome display increased levels of total and non-heme iron in the BAL compared to healthy controls (Ganz, 2009). It is unknown whether increased iron levels in these diseases have a causative effect on lung injury or if the underlying disease-driven oxidative stress leads to the disruption of iron homeostasis. In addition, iron plays a critical role for the survival and proliferation of most pathogens. This fact may have fueled an evolutionary link between iron and immune functions. The host fights infection by sequestering iron in monocytes/macrophages, thus limiting iron availability for extracellular pathogens (Ganz, 2009). This is achieved by recruiting macrophages to the site of infection that withdraw lingering free iron. In addition, as part of the acute phase response, liver-induced hepcidin limits iron export via degradation of ferroportin in iron-exporting cell-types, thus reducing circulating serum iron levels (Nemeth et al., 2004a; Liu et al., 2005; Guida et al., 2015).

In this study, we analyzed a mouse model (*Slc40a1*^C326S/C326S^) with increased iron levels in various tissues, including the lung (Altamura et al., 2014; Neves et al., 2017) where it accumulates mainly in alveolar macrophages, which play a key role in the pulmonary inflammatory response against inhaled pathogens. Iron deposition is further observed in ciliated airway epithelial cells, alveolar type II cells and in a number of vascular smooth muscle cells (Neves et al., 2017). As a model of pulmonary acute inflammation, we administered LPS by nebulization. This approach excludes confounding factors associated with systemic inflammation. Indeed, systemic inflammation was very mild in *Slc40a1*^C326S/C326S^ mice following LPS nebulization, as we observed only a minor induction of liver *hepcidin* mRNA levels (Supplementary Figure 2). Nebulization is also considered superior to intratracheal instillation due to a more uniform distribution of LPS in the lung lobes and a potent inflammatory response at lower doses (Smith et al., 2011; Liu et al., 2013).

Our data demonstrate that iron-overloaded *Slc40a1*^C326S/C326S^ mice show an early attenuated pro-inflammatory signature, as reflected by the decrease in the levels of several cytokines and chemokines associated with monocyte recruitment (*Il1*β, *Il6*, *Cxcl3*, and *Ccl2*; Figure 1). Unexpectedly, reduced cytokine production in *Slc40a1*^C326S/C326S^ mice was not followed by differential neutrophil recruitment to the lungs, a front line response to bacterial infections (Figure 2). Although 4 h after LPS nebulization an altered ratio of neutrophil to macrophage cell populations cannot be detected in the lung of *Slc40a1*^C326S/C326S^ mice (Figure 2B), the mild attenuation of chemokine expression associated with monocyte migration in *Slc40a1*^C326S/C326S^ mice (Figures 1I,J) may suggest potential effects at later time-points when prolonged monocyte accumulation is required for the amplification of the inflammatory response (Maus et al., 2001, 2002) and maximal neutrophil infiltration (Zhang et al., 2020). Future studies will have to assess these responses beyond the acute phase analyzed here. A decreased expression of key cytokines (Figures 1A,B) responsible for stimulating cytokine production in lung cells that do not directly respond to bacterial products may further attenuate the inflammatory response (Toews, 2001). In a recent report by Zhang and coworkers LPS administration by oropharyngeal aspiration in hepcidin KO (knock-out) mice fed with a high-iron diet did not induce differential expression of proinflammatory cytokines. However, the degree of iron overload in the lung, which is far higher in the *Slc40a1*^C326S/C326S^ mouse, as well as the time-point for assessment of cytokine levels may explain the lack of differences observed in LPS administered hepcidin KO mice in comparison to controls (Zhang et al., 2020). The data presented here also contrast previous findings that iron overload causes a pro-inflammatory response of macrophages in other tissues (Zhang et al., 2011; Vinchi et al., 2016; Pereira et al., 2019). The attenuated inflammatory response in LPS-nebulized *Slc40a1*^C326S/C326S^ mice suggests that alveolar macrophages are different in their underlying biology. Previous reports further suggest that iron-loaded macrophages prevent the LPS-mediated pro-inflammatory response by reducing nuclear translocation of the NFκB subunit p65, thereby decreasing expression of target cytokines (Agoro et al., 2018). We extend these observations by demonstrating that not only the expression of the NFκB target cytokines, *Il1*β and *Il6*, but also of the NFκB subunit *RelB*, is attenuated in the lungs of *Slc40a1*^C326S/C326S^ mice aerosolized with LPS. Whether this response reflects the effect of iron overload specifically on alveolar macrophages or other cell types of the lung needs to be dissected in future experiments.

The finding that neutrophil recruitment to the lung of *Slc40a1*^C326S/C326S^ mice after LPS nebulization remains unaltered contrasts previous data obtained in *Hfe*^–/–^ mice, a disease model of the most common form of hereditary hemochromatosis. In *Hfe*^–/–^ mice intratracheal instillation of LPS attenuated recruitment of neutrophils to the bronchoalveolar space, compared to LPS-instilled WT mice, despite increased expression of cytokines/chemokines downstream of TLR4 signaling (Benesova et al., 2012). Differences in the method of LPS administration, the fact that *Hfe*^–/–^ mice do not accumulate iron in the lungs and that alveolar macrophages are iron-depleted may explain model dissimilarities. Furthermore, a specific role for HFE in pulmonary inflammation cannot be excluded. An additional study analyzed hepcidin KO mice that, like *Slc40a1*^C326S/C326S^ mice, display iron overload in the lungs, specifically in alveolar macrophages. Administration of LPS to hepcidin KO mice by intraperitoneal injection, resulted in similar levels of lung myeloperoxidase, commonly used as an indirect measure for the quantification of neutrophils, like in LPS- treated WT mice (Deschemin et al., 2017). Albeit the study in hepcidin KO mice applied LPS via a different route, the findings suggest that alveolar macrophage iron overload does not impair recruitment of macrophages and neutrophils upon acute lung inflammation, e.g., 6-h following an intraperitoneal injection of LPS. Consistent with our data, intranasally LPS infused hepcidin KO and WT mice did not differ in expression of *Cxcl1* and *Cxcl2* (Deschemin et al., 2017), chemokines involved in neutrophil recruitment to the lungs. Importantly, in the study by Zhang et al. (2020) where the same parameters were assessed at later time-points in hepcidin KO mice no differences were observed regarding neutrophil infiltration in the lung upon LPS induction.

Of note is the striking reduction in pulmonary iron levels following LPS exposure of hepcidin KO mice (Deschemin et al., 2017), not observed in *Slc40a1*^C326S/C326S^ and WT mice analyzed in this study (Figure 3A). The decreased pulmonary iron content in hepcidin KO mice could not be explained by differential expression of ferroportin or a decrease of the metal-ion transporters *Zip14* and *Zip8*, which take up NTBI and that are highly expressed in the lung (Wang et al., 2012; Jenkitkasemwong et al., 2015). In this study we observed increased pulmonary mRNA levels of *Zip14*, *Zip8* as well as of *hepcidin*, both in LPS-treated WT and *Slc40a1*^C326S/C326S^ mice (Figures 3C,H,I). However, despite these gene expression changes pulmonary iron levels remained unaltered (Figures 3A,B). This either suggests that an autoregulation of the hepcidin/ferroportin regulatory axis does not exist in the lung or that the kinetics of this inflammatory response are not apparent at the 4 h time-point and that longer LPS exposure times may be required. The latter is supported by preliminary data from bone marrow-derived macrophages differentiated in the presence of Colony stimulating factor 2 (a primary cell model for alveolar macrophages) that display *ferroportin* downregulation upon treatment with TNFα and IL6, after 12 and 24 h, respectively, (data not shown). In line with this, a previous report has also demonstrated *ex vivo* downregulation of ferroportin at the protein level in WT alveolar macrophages stimulated with LPS for 16 h (Nguyen et al., 2006), arguing for longer exposure times to observe a significant decrease in ferroportin *ex vivo* and *in vivo*. In *Slc40a1*^C326S/C326S^ mice the transcriptional downregulation of *ferroportin* would be expected to be the main driving force during inflammation, due to ferroportin resistance to hepcidin (Altamura et al., 2014; Guida et al., 2015).

In conclusion, we show that acute lung inflammation in the context of pulmonary iron overload (Neves et al., 2017) mildly attenuates the inflammatory response to LPS, as reflected by decreased expression of cytokines and chemokines associated with monocyte recruitment. Our findings may be of interest in the context of lung infections suggesting a role of the host’s systemic/lung iron levels in the establishment of the early inflammatory response. As stimulation with LPS may not fully recapitulate a bacterial infection, the influence of lung iron overload upon infection with a proliferating extracellular pathogen remains to be explored in the future and is a subject of public health interest.

## Data Availability Statement

The raw data supporting the conclusions of this article will be made available by the authors, without undue reservation.

## Ethics Statement

The animal study was reviewed and approved by Regierungspräsidium Karlsruhe. Written informed consent was obtained from the owners for the participation of their animals in this study.

## Author Contributions

OM performed experiments, analyzed the data, wrote the original manuscript, and reviewed it. JN designed the experiments, performed the experiments, analyzed the data, wrote the original manuscript, and reviewed it. NH performed experiments and analyzed the data. SA designed the experiments and analyzed the data. MUM designed the experiments, supervised the study, wrote the manuscript, and attained the funding. All authors reviewed and approved the final manuscript.

## Conflict of Interest

The authors declare that the research was conducted in the absence of any commercial or financial relationships that could be construed as a potential conflict of interest.
