# Supplementary material for: Mild Attenuation of the Pulmonary Inflammatory Response in a Mouse Model of Hereditary Hemochromatosis Type 4
Source: Front Physiol. 2021 Jan 13;11:589351. doi: 10.3389/fphys.2020.589351 (PMC7838636; doi:10.3389/fphys.2020.589351)
Supplement: Supplementary file 1 [file Data_Sheet_1.pdf]

Supplementary Material

Table S1. Antibodies used for Flow Cytometry

Antigen	Dilution	Reference
CD45.2	1:200	561096 / BD Biosciences
CD11c	1:200	550261 / BD Biosciences
SiglecF	1:200	552126 / BD Biosciences
Ly-6G	1:100	127605 / BioLegend

Table S2. SYBR-green qRT-PCR primers

	Primer Forward	Primer Reverse
Il1β	GCAACTGTTCCTGAACTCAACT	ATCTTTTGGGGTCCGTCAACT
Il6	GCTACCAAACTGGATATAATCAGGA	CCAGGTAGCTATGGTACTCCAGAA
Tnfα	TGCCTATGTCTCAGCCTCTTC	GAGGCCATTTGGGAACTTCT
Il12β	ATCGTTTTGCTGGTGTCTCC	GGAGTCCAGTCCACCTCTACA

Socs3	CCTTTGACAAGCGGACTCTC	GCCAGCATAAAAACCCTTCA
RelB	CCCGGCACAGCTTTAACAAC	TCTTCAGGGAGCCAGCATTG
Ccl2 (*MCP-1*)	CATCCACGTGTTGGCTCA	GATCATCTTGCTGGTGAATGAGT
Cxcl1 (*KC*)	AGACCATGGCTGGGATTCAC	CGCGACCATTCTTGAGTGTG
Cxcl2 (*MIP-2α*)	CCTGGTTCAGAAAATCATCCA	CTTCCGTTGAGGGACAGC
Cxcl3	CCCCAGGCTTCAGATAATCA	TCTGATTTAGAATGCAGGTCCTT
Ccl3 (*MIP-1α*)	AGATTCCACGCCAATTCATC	GCCGGTTTCTCTTAGTCAGGA
Hepcidin	ATACCAATGCAGAAGAGAAGG	AACAGATACCACACTGGGAA
TfR1	CCCATGACGTTGAATTGAACCT	GTAGTCTCCACGAGCGGAATA
Slc40a1	TGTCAGCCTGCTGTTTGCAGGA	TCTTGCAGCAACTGTGTCACCG

(Fpn)		
Slc39a14 (*Zip14*)	TGGAACCCTCTACTCCAACG	CTGAGGGTTGAAGCCAAAAG
Slc39a8 (*Zip8*)	TCAGCGTTGTATCCCTCCA	GTTTGGGCCCCTTCAGAC
Rpl19	AGGCATATGGGCATAGGGAAGAG	TTGACCTTCAGGTACAGGCTGTG

Table S3. Antibodies used for western blotting

Antigen	Host	Dilution	Reference
TFR1	Mouse	1:1000	136800 / Invitrogen
FTL	Rabbit	1:2500	ab69090 / abcam
FPN	Rabbit	1:500	MTP11-A / Alphadiagnostics
β-actin	Mouse	1:5000	A1978 / Sigma

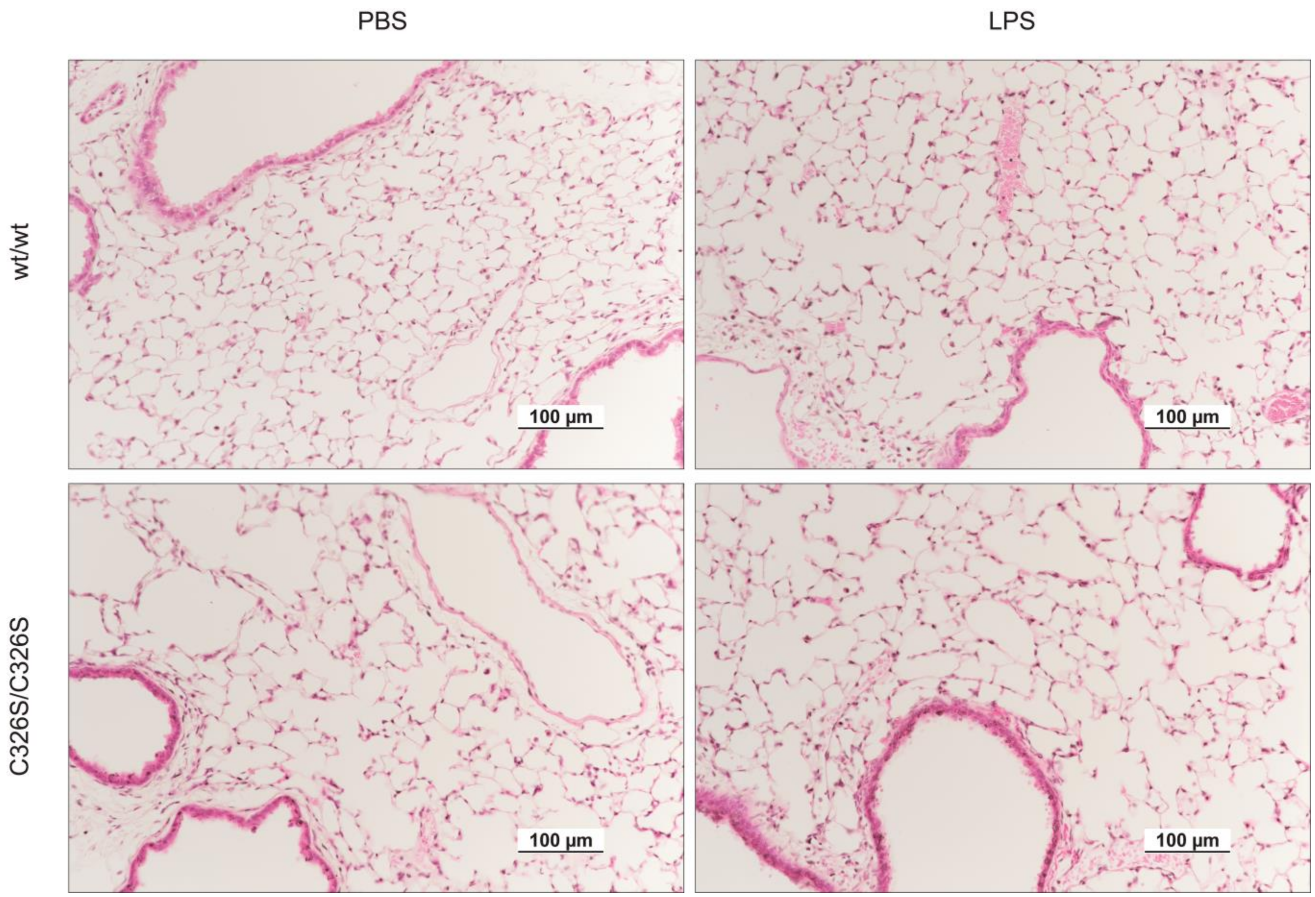

Supplementary Figure S1. Hematoxylin and eosin stain of sections from paraffin embedded lung tissues from 14-week old female wild-type (wt/wt) and *Slc40a1*$^{C326S/C326S}$ (C326S/C326S) mice upon PBS or LPS nebulization.

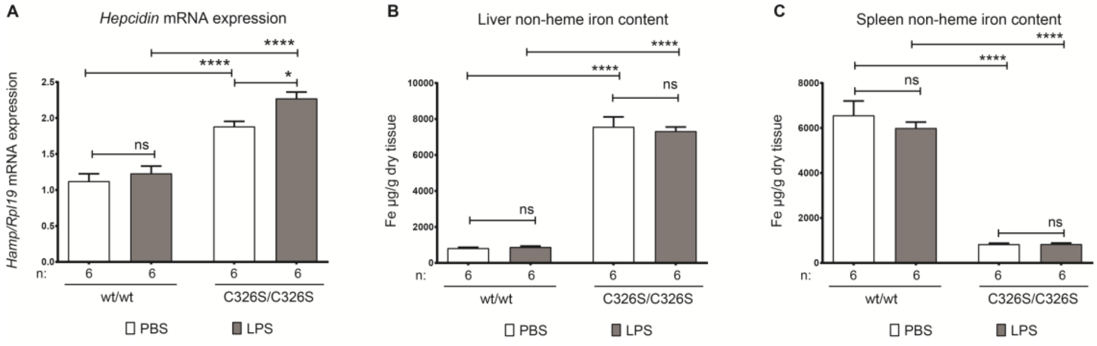

Supplementary Figure S2. Systemic iron homeostasis remains unaffected upon LPS inhalation. (A) qRT-PCR analysis of *hepcidin* in the liver of 14-week old female wild-type (wt/wt) and *Slc40a1*$^{C326S/C326S}$(C326S/C326S) mice upon PBS or LPS nebulization. (B-C) Liver and spleen non-heme iron content of 14-week old female wild-type (wt/wt) and *Slc40a1*$^{C326S/C326S}$(C326S/C326S) mice upon PBS or LPS nebulization. Number of mice analyzed (n) is indicated in each graph. Data are reported as mean ± SEM. Two-way ANOVA: *p < 0.5; ****p < 0.0001.
